# Shifting the paradigm from pathogens to pathobiome: new concepts in the light of meta-omics

**DOI:** 10.3389/fcimb.2014.00029

**Published:** 2014-03-05

**Authors:** Muriel Vayssier-Taussat, Emmanuel Albina, Christine Citti, Jean-Franҫois Cosson, Marie-Agnès Jacques, Marc-Henri Lebrun, Yves Le Loir, Mylène Ogliastro, Marie-Agnès Petit, Philippe Roumagnac, Thierry Candresse

**Affiliations:** ^1^INRA, UMR BIPAR (Enva/Anses) USC INRAMaisons-Alfort, France; ^2^CIRAD, UMR CMAEEPetit-Bourg, Guadeloupe, France; ^3^INRA, UMR 1309 CMAEEMontpellier, France; ^4^INRA, Université de Toulouse, INP, ENVT, UMR 1225, IHAPToulouse, France; ^5^INRA, UMR CBGP (INRA/IRD/Cirad/Montpellier SupAgro)Montferrier-sur-Lez, France; ^6^INRA, Institut de Recherche en Horticulture et Semences, UMR 1345Angers, France; ^7^INRA, UMR 1290, BIOGER-CPP, INRA AgroParis TechThiverval-Grignon, France; ^8^INRA, UMR 1253 STLORennes, France; ^9^Agrocampus Ouest, UMR 1253 STLORennes, France; ^10^INRA, UMR 1333 DGIMIMontpellier, France; ^11^INRA, UMR 1319, MicalisJouy-en-Josas, France; ^12^CIRAD/UMR BGPI TA A54/KMontpellier Cedex 5, France; ^13^UMR 1332 Biologie du Fruit et Pathologie, INRAVillenave d'Ornon Cedex, France; ^14^UMR 1332 Biologie du Fruit et Pathologie, Université de BordeauxVillenave d'Ornon Cedex, France

**Keywords:** next generation sequencing, microbial ecosystem, interactions

## Abstract

The concept of pathogenesis has evolved considerably over recent years, and the scenario “a microbe + virulence factors = disease” is probably far from reality in a number of cases. Actual pathogens have extremely broad biological diversity and are found in all major groups of microorganisms (viruses, bacteria, fungi, protozoa…). Their pathogenicity results from strong and often highly specific interactions they have with either their microbial environment, hosts and/or arthropod vectors. In this review, we explore the contribution of metagenomic approaches toward understanding pathogens within the context of microbial communities. With this broader view, we discussed the concept of “pathobiome” and the research questions that this raises.

## Introduction and research challenges

Recent studies of infectious agents have clearly demonstrated that Koch and Hill's fundamental postulates of “one microbe—one disease” has shown its limits. Indeed, it is now established that many pathogens live and interact with other micro-organisms (not only bacteria, but also protists, fungi, viruses, or phages) in vast communities, all generating and participating in complex interactions that may influence or drive disease processes (Chow et al., 2011; Rogers, 2012). As exemplified by several studies on gut microbial communities, some commensal bacteria of the intestinal flora can become virulent under the influence of diverse factors, such as the actions of other micro-organisms (following horizontal gene transfer between commensal and pathogenic bacteria) (Stecher et al., 2012) or of antibiotics which cause shifts in microbiome composition. Likewise, arthropod vectors carrying human, animal or plant pathogens can be colonized by commensal and mutualistic microbes, which are then able to influence pathogen transmission (Weiss and Aksoy, 2011; Su et al., 2013). For example, it has been shown that the GroEL protein produced by aphid or whitefly endosymbionts is involved in the transmission of plant viruses vectored by these insects (van den Heuvel et al., 1994; Morin et al., 1999) and that symbiotic *Wolbachia* spp. disrupt, by unknown mechanisms, the colonization of mosquito salivary glands by the dengue and Chickungunya viruses, thus limiting their transmission (Tortosa et al., 2008; Mousson et al., 2010, 2012; Blagrove et al., 2013). Similarly, an *Enterobacter* bacterium from the *Anopheles* gut microbial flora renders mosquitoes resistant to infection with the human malaria parasite, by interfering with parasite development through the production of reactive oxygen species (Cirimotich et al., 2011). Such interactions may also exist in other ecosystems (soil, seeds, vertebrate hosts…) and may impact pathogenic processes.

**Figure 1:**
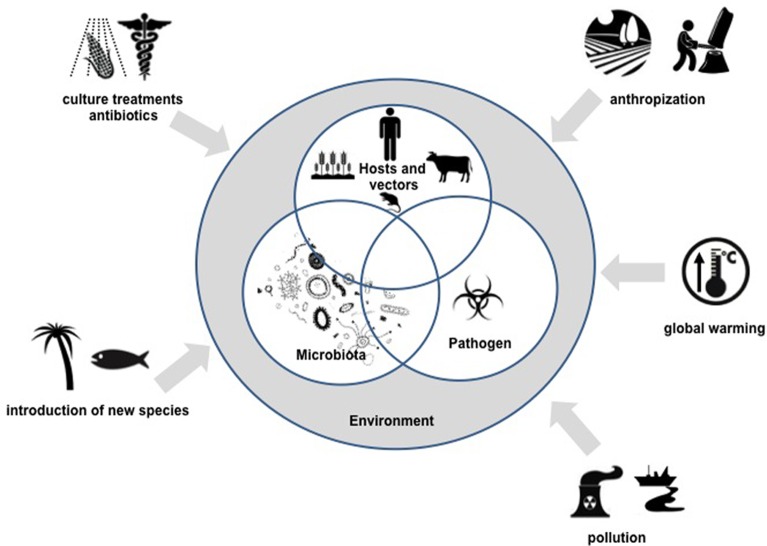
**Overview of the pathobiome concept and scientific challenges for the coming years**.

It is within this context of microbial community interactions, that we define the “pathobiome” concept, which represents the pathogenic agent integrated within its biotic environment. Understanding the pathobiome thus requires (1) an accurate knowledge of the microorganism community defining it, (2) clear evidence of any effect(s) this microorganism community has on pathogenesis, (3) an understanding of the impact of the microorganism community on persistence, transmission and evolution of pathogenic agents, and (4) knowledge of biotic and abiotic factors that may disrupt the pathobiome and lead to onset of pathogenesis. These various aspects represent new scientific issues of remarkable complexity and will constitute major research challenges in the coming years. The development of meta-omics, and in particular metagenomic approaches, will provide invaluable tools in the pursuit of an understanding of pathobiomes.(See Figure 1).

## Research aims

### Inventories to define “pathobiomes” diversity

The first scientific objective is to accurately describe the biodiversity of relevant microbial communities. Toward this goal, major methodological advances are required including the development of new strategies, techniques and tools to achieve an effective and comprehensive inventory and description of pathobiome diversity (Zaura, 2012; Frisli et al., 2013). Recent advances in high throughput sequencing technologies (e.g., 454 and Illumina) enable the exploration of microbial diversity at an unprecedented scale. However, novel methodologies are now required to analyze this wealth of data. Indeed, metagenomic studies have revealed the extraordinary richness and diversity of viral, bacterial and parasitic communities in multiple different ecosystems (Willner et al., 2009; Santos and Anton, 2011; Fancello et al., 2012; Lecuit and Eloit, 2013). However, the tools required to accurately measure and compare pathobiome diversity, such as bioinformatic pipelines and databases, are still largely rudimentary. In order to measure the impact of the community on the occurrence, transmission, and evolution of a given pathogen, every pathogen- and/or pathobiome-specific microbial community from diverse biological and/or environmental samples will need to be described and compared. In addition to serving as a starting point to address questions in other research areas, these approaches will have direct impact on the broad fields of diagnosis and etiology (Barzon et al., 2011; Chiu, 2013; Sherry et al., 2013). For the first time, these methods will permit reliable and sensitive identification of pathogens associated with each ecosystem (such as within a plant compartment, an animal organ or an arthropod vector) without any *a priori* knowledge. As with other metagenomic studies, it is clear that these approaches will reveal hitherto unsuspected microorganisms, including novel pathogens, as has already been demonstrated in several studies (Chiu, 2013; Beer et al., 2013; Drexler et al., 2013).

For some methodological aspects, it will be possible to rely on developments from more advanced fields of metagenomics, such as environmental microbiology (Rajendhran and Gunasekaran, 2008; Buee et al., 2009; Lombard et al., 2011). This is indeed the case for the bioinformatic analysis of bacterial 16S data, fungal ITS data or metatranscriptomic approaches developed for other microbial ecosystem studies (Cole et al., 2009; Schoch et al., 2012; Sherry et al., 2013). The increasingly widespread multiplexing approaches are also an interesting and affordable way to generate the large amounts of data necessary for the application of comparative statistical approaches to microbial communities from numerous samples (See Cosson et al., in this issue).

Certain specific aspects of the pathobiome will require tailored and original developments, such as the ability to identify pathotypes and genotypes to an infra-specific level, and will need to address the additional specific problems generated by phages, viruses, and parasites.

Finally, it should be emphasized that we still only have a very limited understanding of how pathogenic agents themselves, including bacteriophages (as pathogens of bacteria), contribute to the functioning of many ecosystems (Santos and Anton, 2011), so that this aspect will undoubtedly be an important issue to be addressed in the coming years.

### Pathobiome modulation of pathogenesis, functional impact of the community

A second major challenge is to shift from descriptive to functional metagenomics, in order to understand exactly how the pathobiome modulates pathogenesis, and to which extent it impacts on its environment, regardless of the ecological niche considered. A large number of studies have been dedicated to understanding how a single pathogen interacts with its host, without taking into account the role of the overall microbial environment. The transition to a broader pathobiome perspective involves revisiting the Koch and Hill's postulates at the level of microbial communities, and raises the question of the nature and diversity of the microbiota present in a healthy individual, and of factors promoting the emergence of potentially disease-causing pathobiomes. For instance, as recently reviewed (Lecuit and Eloit, 2013), the role of the gut microflora community is becoming increasingly recognized as a key factor in the onset of inflammatory diseases. Similarly, the microbiota contribution toward promoting infection and spread of enteric viruses is receiving more attention (Kuss et al., 2011). In the future, understanding the interaction of the microbiota with pathogens (=pathobiome) and the host might provide new insights into the pathogenesis, as well as novel avenues for preventing and treating intestinal and systemic disorders.

The spreading of antibiotic resistance genes is another example of the vital importance of the microbial context, as pathogens may be able to acquire resistance phenotypes from environmental reservoirs (Dantas and Sommer, 2012; Perry and Wright, 2013). The most recent evidence for the transfer of antibiotic resistance genes between the environment and the clinic is provided by Forsberg et al. (2012). Using an innovating metagenomic approach, the authors identified DNA sequences in cultured multi-drug-resistant soil Proteobacteria which had 100% identity to resistance genes found in clinical pathogens, including resistance against β-lactams, tetracyclines, aminoglycosides, sulfonamides, and chloramphenicol. Although the authors could not definitively show that these genes originated from soil organisms due to the nature of their metagenomic approach, these results emphasize the importance of the soil resistome, regardless of the direction of gene flow (from soil to clinic, or vice versa). Functional metagenomic approaches offer an opportunity to unravel the role of the host in shaping and modulating the equilibrium of these microbial communities. The host's response(s) and their “history” (etiological, epidemiological, immunological) will also need to be taken into account (Conrath et al., 2002).

Because metagenomic DNA-based analyses cannot differentiate between expressed and non-expressed genes, these approaches fail to reflect the actual activity or dynamics of microbial communities. The need to develop broader “meta-omic” approaches such as *in situ* metatranscriptomics and metaproteomics is thus clear as any technical limitations of these methods are being progressively circumvented (i.e., by direct next-generation cDNA sequencing for transcriptomics) (Simon and Daniel, 2011). A metatranscriptomic study conducted on a multispecies biofilm model composed of species found in healthy oral biofilms has, for example, recently unraveled the importance of pathogens in controlling gene expression of a healthy oral community, thus demonstrating the usefulness these approaches (Frias-Lopez and Duran-Pinedo, 2012).

Short term priorities remain the acquisition of metagenomic data with improved quality from ecosystems of interest (sampling of relevant pathobiomes). To address and resolve the complexity of these systems, innovative strategies are needed that will require in-depth mathematical and statistical analyses.

Functional metagenomic approaches will also aim to determine the common denominator as well as the specificities of particular ecosystems. Indirectly, these methods raise the question of the nature of the “functional biological model” studied for a given pathobiome, and of its integrative scale, from single cell to complex populations, including their intricate intermediate levels (tissues, organs, individuals…). Experimental designs must be carefully prepared and implemented such as to mimic these complex interactions, continually taking into account co-infection and synergies between different microorganisms. Here, the challenge will be to identify those pathobiome agents which are beneficial to the host (probiotics, bacteriophages, competitors, activators of host defences), the conditions that may hinder the development of pathogenesis, or the emergence of damaging variants or sub-populations from healthy communities (Santos and Anton, 2011; Zarco et al., 2012).

### Microbial ecology at the level of the pathobiome

The third goal is to understand the impact of microbial communities on the persistence, transmission, and evolution of pathogens. In particular, to elucidate the spatio-temporal dynamics of these communities, the ecological interactions between microorganisms within communities, as well as the evolutionary forces at play (mutation, selection and gene flow)—especially when it comes to pathogen survival, extinction or dispersal. Many macro-ecological concepts could be applied to micro-ecology, although some may require reformulation to fit the microbial lifestyle (Little et al., 2008; Rogers et al., 2013).

The ecosystems within which pathogens evolve are defined by their abiotic and biotic dimensions, including both host and vector populations. Pathogens must adapt to selective pressures associated with these complex ecosystems, in particular to both host and vector defense mechanisms (Little et al., 2008; Rogers et al., 2013), as well as to any environmental fluctuations impacting on their survival and/or dispersal outside of hosts or vectors (see next section). In addition, microorganisms interact with each other. Antagonistic and mutualistic behaviors have evolved as adaptations to living in microbial communities. Microorganisms cooperate, compete with, or even prey upon each other to better exploit resources. As with macroorganisms, competition for nutrients and space plays an important role in shaping microbial interactions. In the human gut for instance, competition for resources is thought to impede pathogen infection, a phenomenon called the “barrier effect” (Casadevall and Pirofski, 1999; Akira et al., 2006; Jones and Dangl, 2006). Such a barrier effect has also been implicated in counteracting pathogen infection at the epidermal surface of both plants and animals (Beattie and Lindow, 1999; Chen and Tsao, 2013; Findley et al., 2013). Antagonistic effects have also been evidenced in the mosquito midgut between the bacteria *Enterobacter* sp. and the human malaria parasite, *Plasmodium falciparum*, leading to reduced malaria transmission by mosquitoes (Cirimotich et al., 2011). The killing of bacteria by microbial eukaryotes and bacteriophages also has a global effect on bacterial communities (Pernthaler, 2005). In the field of environmental science, for example, phages have been shown to play a crucial role in structuring microbial communities involved in biogeochemical cycling (Suttle, 2007). A better understanding of phage–host interaction has already benefited those secondary applications which rely on phage-resistant bacteria to produce foods and biotechnological products (Samson and Moineau, 2013). Although similar studies in the field of therapeutics are lacking, it is also hoped that knowledge of phage–bacterium dynamics will eventually contribute to our ability to manipulate our own microbiota by using adapted phages as biocontrol agents. Within this context, the use of phage therapy has recently received renewed interest in an effort to solve those problems in human therapy linked to antibacterial resistance (Samson et al., 2013).

Individual microorganisms evolve in the context of a community. In many ways, the evolution of a microorganism is influenced by selection pressures exerted by other microorganisms in the community. Mutation and gene flow are responsible for the creation of new genetic variants within populations. In addition, natural selection and genetic drift act by maintaining, increasing, or decreasing the frequency of these new genetic variants within populations. These processes shape the genetic composition of bacterial communities as well as their functional properties. The transfer of genetic information between species is a central and original mechanism of generating genetic diversity in microbial communities (Hacker and Kaper, 2000; Ochman et al., 2000). For example, bacterial species can exchange large regions of DNA potentially linked to pathogenicity (Censini et al., 1996) as exemplified with *Edwardsiella tarda*, an enterobacterium causing fatal disease in fish. Genomic comparison between pathogenic and non-pathogenic strains indicated that disease-causing strains possessed two pathogenic islands of Type VI and Type III secretion systems, which were absent in harmless strains. The Type III secretion system is homologous to the enterocyte effacement locus in enteropathogenic and enterohemorrhagic *Escherichia coli*. Evolutionary analysis indicated that this locus was integrated into the *E. tarda* genome through horizontal transfer from *E coli* (Nakamura et al., 2013). The architecture of communities, which dictates the phylogenetic proximity of microbes, affects the probability of gene exchanges. A trait can thus sweep through a microbial community under appropriate selection pressure, resulting in strong functional implications, such as pathogenicity or the capacity to infect different host species as has been recently shown for *Bartonella* spp. (Guy et al., 2013).

A major challenge in pathobiome research will thus be to develop and apply concepts and approaches of microbial ecology and evolutionary biology to pathogens and the microbial communities in which they exist. A peculiarity of pathobiomes is that they involve multiple complex interactions within microbial communities, the host and the environment, and are subject to strong selection pressures that sometimes lead to extreme adaptations (e.g., species which have recently become intracellular parasites, (Casadevall, 2008). Metagenomic approaches will facilitate the complete description of microbial communities associated with the different phases of a pathogen's life cycle, and thus will aid in the identification of species that may act as partners or antagonists. Particular attention should be paid to the impact of the pathobiome on the molecular interactions involved in infection and transmission (vertical or horizontal). To effectively combat plant and animal diseases, a major challenge will be to identify those levers which control or manipulate those microbial communities associated with pathobiomes. Understanding the internal processes of microbial communities is necessary to enable predictive modeling of ecological dynamics of microbial communities. Such predictive models can provide guidance for strategies aiming to manipulate communities in plants or animals and modulate their pathogenicity.

### Selective forces, such as environmental and human-mediated disturbances, shape viral, and bacterial pathobiomes

The emergence of socially/economically relevant diseases is likely to be frequently linked with ecological disturbances caused by human intrusions into natural ecosystems (Anderson et al., 2004; Jones et al., 2008). Displacement of natural plant and animal species by intensive agriculture (Malmstrom et al., 2005a) or anarchic urban developments, including disturbances associated with unmanaged sewage (Dinsdale et al., 2008), pollutants and/or radioactivity, are all important subsets of those intrusions that represent fundamental disturbances in the diversity and in the evolutionary dynamics of viral and bacterial communities inhabiting natural ecosystems. For instance, studies have shown that invasive exotic annual grasses can indirectly increase barley and cereal yellow dwarf virus (B/CYDVs) disease incidence in California native perennial bunchgrasses (Malmstrom et al., 2005b). Beyond this example, the numbers of situations in which introduced domesticated plants/animals come into contact for the first time with indigenous viruses or bacteria from native plants/animals, have dramatically increased alongside the intensification of human activities.

Molecular biology techniques such as molecular typing and, more recently, metagenomics, have led to considerable progress in understanding adaptive responses of pathogen populations to human-mediated disturbance. For instance, studies of *Staphylococcus aureus* populations have provided examples of how human activities can change a population structure, inducing adaptation of the pathogen to a new host species. Comparative genomics clearly links genomic- and phenotypic-characteristics with host adaptation, and reveals that major animal-associated clones most likely emerged after a human-to-animal host jump, followed by subsequent adaptive evolution. *S. aureus* transmission from human to bovine hosts was thought to have occurred ~5500 years ago, during the Neolithic revolution and the expansion of agriculture throughout the Old World (Weinert et al., 2012). Similarly, the current predominantly poultry-associated clones have most probably emerged after a human-to-poultry host jump (Lowder et al., 2009), estimated to have occurred ~50 years ago (Weinert et al., 2012), and which rapidly disseminated worldwide thanks to the globalization of the poultry industry (Lowder et al., 2009; Fitzgerald, 2012).

Strong environmental pressures can also shape pathobiomes, and turn otherwise pathological interactions into mutualistic relationships. In extreme environments, such as in geothermal soils in Yellowstone National Park, evolution has selected plants that harbor fungal endophytes infected with a virus, and all three partners are required for thermal tolerance of the system (Marquez et al., 2007).

Classical molecular biology approaches have shed light on the role of environmental and human-mediated constraints on single microbe species or very limited groups of species, but cannot fully account for the structure and evolution of entire pathobiomes in response to selective pressures. Pioneering studies have recently generated insights into interactions occurring within the pathogen-host-microbiome “ménage à trois” (Pedron and Sansonetti, 2008) but in-depth analyses are necessary to better understand the impact of selective pressures on the interactions between the three players, which can result in the pathogen acquiring new functions by horizontal gene transfer or convergent evolution. These interactions may ultimately lead to the occurrence of new, more virulent variants, or even to the emergence of new diseases due to the combination of synergistic/antagonist phenomena between different pathogens and non-pathogens (fungal, bacterial, or viral) in mixed or reconstructed communities. In contrast, the acquisition of new functions by the pathogen may reduce pathogenesis or even increase host tolerance, resulting in a shift toward mutualism. This has been demonstrated for several acute viruses inducing drought tolerance, an important trait in a changing environment, or for the *Cucumber mosaic virus* which confers cold tolerance in red beets (Xu et al., 2008).

Thus metagenomics might help decipher the mechanisms involved in pathobiome evolution in response to multiple factors:

- host factors (e.g., host diet, immune status, host jumps, changes in vectors/reservoirs, etc.),
- human activity driven factors, such as implemented control strategies (e.g., culture or seed treatments, antibiotic prophylaxis, etc..), as well as cropping practices or breeding conditions, which can all directly impact both pathogens and the ecosystem in which they reside,
- environmental factors (e.g., global warming, abiotic stress imposed on the host(s), vector(s) or reservoir(s), location of the host(s) in developed or wild areas etc.).

A better understanding of the forces structuring pathogen populations (including pathogen plasticity and their interactions with the host and its microbiota) have the potential to rejuvenate control strategies aiming for better efficacy against the target pathogen(s).

## Barriers and constraints to pathobiome studies

The barriers to the development of pathobiome studies are both methodological (most likely common to other ecosystems) and conceptual. Regarding the methodological barriers, they pertain to:

- the sampling and sample treatment strategies used to obtain reliable qualitative and quantitative inventories of microbial diversity in biological samples (plants, animals, arthropods or other relevant ecosystems); the unbiased purification and amplification of nucleic templates (DNA, RNA) (Kim and Bae, 2011), and the preparation of balanced pools during multiplexing. Targets for sequencing will be microorganism-dependent, with different and specific problems linked to viruses, bacteria, and parasites.
- the scaling up of these methods for deeper sequencing, and/or the analysis of larger numbers of biological samples obtained from contrasting environmental conditions to achieve adequate coverage and unbiased microbe identification (Fichot and Norman, 2013; Wendl et al., 2013), to allow comparative analyses, and to identify factors that structure microbial communities. Thorough comparative analyses require that sample are identified through a standard for minimum information (Yilmaz et al., 2011).
- bioinformatic data analysis: many bioinformatic approaches and tools are available (software, computing platforms, and pipelines), (Prakash and Taylor, 2012) but so far any real comparative evaluation of these tools or concerted efforts toward their improvement are still largely lacking. In addition, these tools are often also poorly accessible to pathology laboratories, which are traditionally focused on single agent issues, particularly in project development stages. Finally, long-term data storage and communication/collaboration/datasharing issues remain of importance.
- tool development for functional pathobiome analysis, in particular the statistical analysis of relationships between the pathobiome and pathogenesis. Comprehensive metadata analysis, mathematics, and statistics are required to extract the relevant information.
- modeling approaches to analyze the interactions between microorganisms, and their impact on pathogen transmission are still lacking.

In parallel with these methodological barriers, researchers interested in the “pathobiome” face conceptual hurdles which must be overcome. New sequencing technologies have shifted the study focus from the organism to the community of pathogens and other microbes within their environment. Such an expanded point of view results in a paradigm shift, in that the “pathogen” is no longer understood to be a single isolated organism. It is therefore vitally important to integrate ecological and evolutionary concepts of microbial communities to be able to fully grasp the roles that ecosystem composition and structure play in the emergence of pathogens and the expression of virulence (Rogers et al., 2013). A key barrier thus relates to the “pathologists” appropriation of microbial ecology concepts and approaches.

## Perspectives

Three major research themes were identified over and above developing methodological approaches and discovering new human and plant disease-causing pathogens: (1) investigating the contribution of the biotic environment or pathobiome to the onset and progression of pathogenicity, (2) understanding the community ecology of pathogens, and (3) elucidating the forces and mechanisms which structure over time and space the communities in which pathogens evolve. In each of these areas, the first step will be to overcome a number of methodological barriers (in particular, but not only in terms of bioinformatic analysis) in order to generate accurate metagenomic inventories and descriptions of pathobiomes diversity. An additional hindrance when attempting to compare metagenomes, is the limited submission of metagenomic data to publicly accessible databases, as it is currently not mandatory when publishing results.

Finally, a major conceptual issue concerns the gap between the “pathogen focused” culture of a vast majority of pathologists, and the ecological concepts necessary to tackle microorganism communities as a whole. In the future, the only way to successfully address these new and exciting issues will be to ensure that researchers remain open to these new concepts, facilitating valuable translational collaborations and exchanges between pathologists and ecologists.

### Conflict of interest statement

The authors declare that the research was conducted in the absence of any commercial or financial relationships that could be construed as a potential conflict of interest.
